# Associations between the Platelet Lymphocyte Ratio and Albumin with Plaque Calcification in Patients with Acute Coronary Syndrome: An Optical Coherence Tomography Study

**DOI:** 10.31083/RCM28167

**Published:** 2025-04-27

**Authors:** Kexin Song, Wenjing Yao, Haihao Yan, Yan Zhang, Yanhong Li, Tianxing Li, Qian Yang, Feifei Zhang, Yi Dang

**Affiliations:** ^1^Department of Graduate School, Hebei Medical University, 050017 Shijiazhuang, Hebei, China; ^2^Department of Cardiology Center, Hebei General Hospital, 050051 Shijiazhuang, Hebei, China; ^3^Tianjin Union Medical Center, Tianjin Medical University, 300122 Tianjin, China; ^4^Department of Graduate School, Hebei North University, 075000 Zhangjiakou, Hebei, China

**Keywords:** acute coronary syndrome, coronary artery calcification, optical coherence tomography, platelet to lymphocyte ratio, albumin, malnutrition, inflammation

## Abstract

**Background:**

Coronary artery calcification (CAC) is a robust independent predictor of cardiovascular events. Therefore, it is essential to elucidate the factors that influence CAC progression to enhance the outcomes of patients diagnosed with acute coronary syndrome (ACS). This study aimed to investigate the relationship between prevalent laboratory parameters and the calcification of coronary artery plaques in patients diagnosed with ACS by applying optical coherence tomography (OCT).

**Methods:**

This single-center, cross-sectional study retrospectively evaluated patients with ACS who underwent percutaneous coronary intervention and OCT examinations at the Hebei General Hospital. Baseline data, laboratory parameters, and OCT imaging were analyzed. Comprehensive statistical analyses were conducted to elucidate the relationship between prevalent laboratory parameters and coronary artery plaque calcification.

**Results:**

In this study involving 130 patients, the platelet to lymphocyte ratio (PLR) demonstrated a significant positive correlation with coronary artery plaque calcification (r_s_ = 0.373, *p* < 0.001), whereas albumin exhibited a significant negative correlation (r_s_ = –0.585, *p* < 0.001). Both the PLR (odds ratios (OR) 1.011, 95% CI 1.002–1.019, *p* = 0.014) and albumin levels (OR 0.642, 95% CI 0.539–0.764, *p* < 0.001) emerged as significant independent predictors of plaque calcification. Receiver operating characteristic curve analysis identified a cutoff point for albumin at <40.65, yielding a sensitivity of 75.8% and a specificity of 77.9%, Comparatively, a PLR >145.04 demonstrated a sensitivity of 61.3% and a specificity of 76.5% for predicting plaque calcification.

**Conclusions:**

Albumin and the PLR were significantly associated with plaque calcification in patients with ACS, serving as independent predictors of coronary artery plaque calcification. These parameters may significantly contribute to risk stratification and the future development of preventive strategies to mitigate adverse cardiovascular events.

## 1. Introduction

Since 1921, heart disease has consistently emerged as a leading cause of mortality in the United States. Although mortality rates associated with cardiovascular diseases have shown a decline in recent years, the incidence rate reveals an upward trajectory [1]. Despite the widespread adoption and significant advancements in vascular revascularization procedures and secondary preventive medications, patients diagnosed with coronary artery disease (CAD) continue to represent a high-risk population for recurrent cardiovascular events, especially those individuals with a history of acute coronary syndrome (ACS) [2]. Estimates indicate that over seven million individuals globally receive a diagnosis of ACS each year [3]. The predominant cause of ACS is the rupture of atherosclerotic plaques, which subsequently leads to the formation of secondary thrombi [3]. The presence and extent of coronary artery calcification (CAC) serves as direct indicators of the presence and severity of CAD and are closely linked to plaque progression and instability [4, 5]. Furthermore, the ability of CAC to predict future cardiovascular events, independent of traditional risk factors, surpasses that of any other non-invasive biomarker [6]. A prospective, Multi-Ethnic Study of Atherosclerosis (MESA) demonstrated that CAC is significantly associated with a 10-year risk of future cardiac events [7]. CAC presents a substantial challenge to percutaneous coronary intervention (PCI). Extensive CAC significantly impairs the effectiveness of balloon angioplasty and obstructs both stent delivery and expansion [8]. Regardless of the technique employed, PCI in heavily calcified coronary vessels may elevate the risk of no-reflow, coronary artery entrapment, stent damage or dislodgement, and periprocedural myocardial infarction (MI) [9]. Consequently, the investigation of factors associated with the occurrence and progression of CAC, with the objective of delaying the advancement of coronary atherosclerosis, is of paramount clinical importance for reducing both readmission and mortality rates among patients with ACS. Coronary angiography is routinely utilized to evaluate the extent and severity of arterial stenosis. Although coronary angiography is capable of detecting a broad spectrum of superficial calcified plaques, its sensitivity for smaller lesions is less than 50% and is contingent upon the operator’s proficiency [10]. Optical coherence tomography (OCT), recognized as a high-resolution cross-sectional imaging modality within the coronary arteries, facilitates the precise identification of internal structures and plaque characteristics, and has significant potential for delineating the intricate details of CAC [11]. Given the critical role of inflammation in atherosclerosis, we first hypothesized that inflammation-related biomarkers are associated with CAC in patients with ACS and then sought to evaluate the potential relationship between prevalent laboratory parameters and coronary artery plaque calcification through the use of OCT.

## 2. Materials and Methods

### 2.1 Study Population

In this single-center, cross-sectional study, we retrospectively sought to evaluate the potential relationship between prevalent laboratory parameters and coronary artery plaque calcification through the application of OCT, while also exploring novel risk and protective factors to enhance the efficacy of secondary prevention strategies and mitigate the malignant progression of coronary artery plaques. Patients diagnosed with ACS [12] who underwent direct PCI and OCT examinations at the Hebei General Hospital between January 2019 and December 2023 were included in the study. Patients excluded from the study (n = 23) had undergone previous revascularization procedures, presented with cardiogenic shock, severe hepatic or renal dysfunction, left main coronary artery narrowing, or had severely tortuous vessels. OCT images from 155 enrolled patients with ACS were initially analyzed, with 25 patients subsequently excluded due to poor image quality (n = 6), in-stent restenosis (n = 9), or pre-dilatation before OCT imaging (n = 10). Final OCT images from 130 eligible patients with ACS were included in the analysis.

### 2.2 Baseline Data Collection

Patient demographics, laboratory results, and clinical data were meticulously extracted from the medical records system. Laboratory results including hemoglobin, white blood cell, neutrophil, lymphocytes, platelets, platelet to lymphocyte ratio (PLR), albumin, fasting blood glucose, uric acid, estimated glomerular filtration rate, total cholesterol, triglyceride, low-density lipoprotein cholesterol, high-density lipoprotein cholesterol, lipoprotein(a), apolipoprotein A1, and apolipoprotein B. Upon hospital admission, peripheral venous blood specimens were collected for comprehensive hematological profiling using our institution’s laboratory facilities. Additional peripheral venous blood samples were obtained the following morning after an overnight fast for the assessment of biochemical parameters. Clinical data including heart rate, systolic blood pressure, diastolic blood pressure, type of ACS, angiographic findings, hypertension, diabetes mellitus, hyperlipidemia, atrial fibrillation (AF), family history of CAD, and smoking status were recorded. Patients who were actively smoking or had ceased smoking for less than one year at the time of admission were classified as current smokers. Body mass index (BMI) was calculated by dividing weight (kilograms) by height (meters) squared.

### 2.3 Optical Coherence Tomography Procedure and Imaging Analysis

**Fig. 1.**
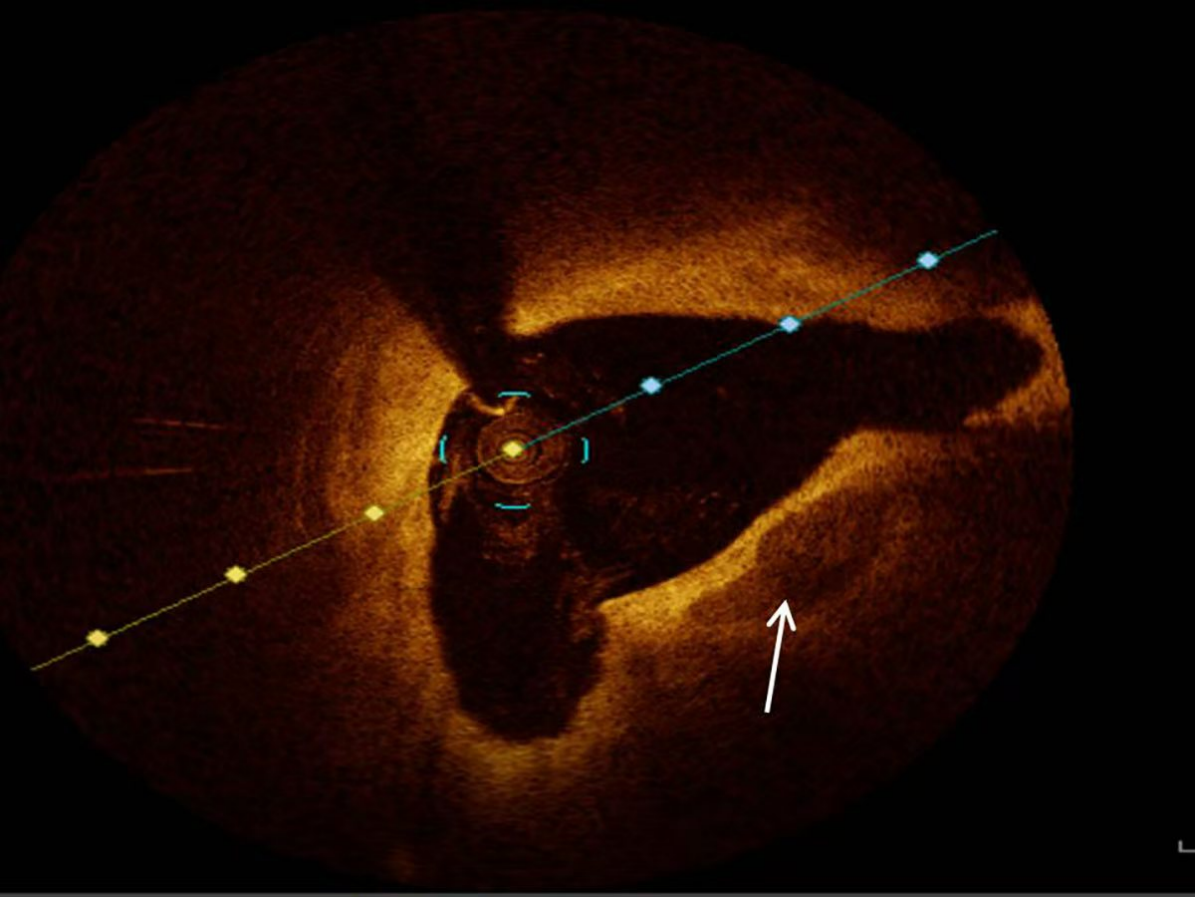
**Representative optical coherence tomography image of calcification (white arrow)**.

All patients diagnosed with ACS underwent OCT examinations utilizing the ILUMIEN OPTIS system (Abbott Vascular, Santa Clara, CA, USA) for imaging. The OCT data were interpreted and documented by two experienced interventional cardiologists who remained blinded to the clinical and laboratory data of the study population. In instances of disagreement regarding the interpretation of OCT images, resolution was achieved through consultation with an additional experienced interventional cardiologist. The characteristics of coronary artery plaques were documented in accordance with established guidelines derived from previous studies and consensus standards [13]. Calcified plaques presented as distinct areas characterized by well-defined borders, low attenuation, and heterogeneous low signal intensity [10, 13] (Fig. 1).

### 2.4 Statistical Analysis

Continuous variables were presented as the mean ± standard deviation. Normally distributed continuous variables were compared using the Student’s *t*-test, whereas non-normally distributed variables were analyzed using the Mann-Whitney U test. Categorical data were expressed as frequencies (percentages) and compared using Pearson’s chi-square test, the continuity-corrected chi-square test, or Fisher’s exact test. Spearman rank correlation analysis was employed to investigate the relationship between the PLR and albumin levels in relation to coronary artery plaque calcification. Predictive factors for coronary artery plaque calcification were evaluated using both univariate and multivariate logistic regression analyses (conducted using the Enter method) parameters exhibiting a *p* < 0.05 in the univariate analysis were incorporated into the multivariate model. A receiver operating characteristic (ROC) curve was constructed to assess the predictive value of the PLR and albumin levels for coronary artery plaque calcification, including the calculation of the area under the ROC curve (AUC) and its corresponding 95% confidence interval. Statistical analyses were performed using SPSS version 26.0 (IBM, Armonk, NY, USA). A significance level of two-sided *p* < 0.05 was deemed statistically significant.

## 3. Results

### 3.1 Baseline Characteristics

In total, 130 patients who satisfied the inclusion criteria were enrolled in this study. Based on the presence or absence of plaque calcification, the enrolled patients were categorized into the non-plaque calcification group (n = 68) and the plaque calcification group (n = 62). The baseline clinical characteristics and laboratory parameters for both groups are presented in Tables 1,2. The mean age of the study population was 52.30 ± 14.81 years, with 89.2% of participants identifying as male. Laboratory parameters are described in Table 2. The plaque calcification group exhibited a significantly lower lymphocyte count (1.61 ± 0.69 vs. 2.18 ± 1.17, *p* = 0.002) and albumin levels (38.28 ± 4.07 vs. 43.45 ± 3.66, *p* < 0.001) compared to the non-plaque calcification group. Furthermore, the PLR (176.92 ± 81.18 vs. 126.40 ± 55.04, *p* < 0.001) was significantly higher in the plaque calcification group than in the non-plaque calcification group (*p* < 0.001). Other parameters demonstrated no statistically significant differences between the two groups (*p* > 0.05).

**Table 1.** **Baseline characteristics**.

Parameters		Total (n = 130)	Non-plaque calcification (n = 68)	Plaque calcification (n = 62)	*p* value
Age, years		52.30 ± 14.81	52.31 ± 15.59	52.29 ± 14.04	0.994^#^
Male, n (%)		116 (89.2)	60 (88.2)	56 (90.3)	0.701
BMI (kg/m^2^)		25.54 ± 2.81	25.73 ± 3.12	25.34 ± 2.45	0.734
Heart rate (beats per minute)		72.56 ± 13.07	73.99 ± 13.86	71.00 ± 12.06	0.296
SBP (mmHg)		133.38 ± 20.70	136.66 ± 19.48	129.79 ± 21.55	0.065
DBP (mmHg)		83.58 ± 15.03	85.47 ± 14.92	81.52 ± 14.99	0.135^#^
LVEF (%)		59.32 ± 7.70	59.69 ± 8.49	58.90 ± 6.77	0.300
Type of ACS, n (%)					0.721
	STEMI	63 (48.5)	33 (48.5)	30 (48.4)	
	Non-STEMI	12 (9.2)	5 (7.4)	7 (11.3)	
	UAP	55 (42.3)	30 (44.1)	25 (40.3)	
Culprit vessels					0.020
	LAD	81 (62.3)	49 (72.1)	32 (51.6)	
	LCX	8 (6.2)	5 (7.4)	3 (4.8)	
	RCA	41 (31.5)	14 (20.6)	27 (43.5)	
Coronary artery lesions					0.437
	SVD	77 (59.2)	41 (60.3)	36 (58.1)	
	DVD	35 (26.9)	20 (29.4)	15 (24.2)	
	TVD	18 (13.8)	7 (10.3)	11 (17.7)	
Pre-TIMI flow					0.634
	0	32 (24.6)	17 (25.0)	15 (24.2)	
	1	5 (3.8)	2 (2.9)	3 (4.8)	
	2	9 (6.9)	3 (4.4)	6 (9.7)	
	3	84 (64.6)	46 (67.6)	38 (61.3)	
Hypertension, n (%)		62 (47.7)	34 (50)	28 (45.2)	0.581
Diabetes mellitus, n (%)		31 (23.8)	16 (23.5)	15 (24.2)	0.929
Hyperlipidemia, n (%)		28 (21.5)	16 (23.5)	12 (19.4)	0.563
Atrial fibrillation, n (%)		5 (3.8)	2 (2.9)	3 (4.8)	0.916
Family history of CAD, n (%)		21 (16.2)	13 (19.1)	8 (12.9)	0.336
Current smoking, n (%)		75 (57.5)	36 (52.9)	39 (62.9)	0.251

Continuous data are presented as mean ± standard deviation. Categorical data are presented as number (%). BMI, body mass index; SBP, systolic blood pressure; DBP, diastolic blood pressure; LVEF, left ventricular ejection fraction; ACS, acute coronary syndrome; STEMI, ST-segment elevation myocardial infarction; UAP, unstable angina pectoris; LAD, left anterior descending artery; LCX, left circumfex artery; RCA, right coronary artery; SVD, single vessel disease; DVD, double vessel disease; TVD, triple vessel disease; pre-TIMI, previous of procedural thrombolysis in myocardial infarction flow grade; CAD, coronary artery disease. For continuous variables, “^#^” denotes comparisons made using the Student’s *t*-test, while other variables were compared using the Mann-Whitney U test.

**Table 2.** **Laboratory parameters**.

Parameters	Total (n = 130)	Non-plaque calcification (n = 68)	Plaque calcification (n = 62)	*p* value
Hemoglobin (g/L)	145.29 ± 16.13	147.15 ± 15.16	143.26 ± 17.02	0.171^#^
WBC (×10^9^)	7.78 ± 1.92	7.67 ± 1.81	7.91 ± 2.03	0.403
Neutrophils (×10^9^)	5.03 ± 1.55	4.86 ± 1.41	5.21 ± 1.69	0.190^#^
Lymphocytes (×10^9^)	1.91 ± 1.01	2.18 ± 1.17	1.61 ± 0.69	0.002
Platelets (×10^9^)	240.78 ± 64.47	232.76 ± 52.29	249.58 ± 75.09	0.429
PLR	150.49 ± 73.02	126.40 ± 55.04	176.92 ± 81.18	0.000
Albumin (g/L)	40.98 ± 4.63	43.45 ± 3.66	38.28 ± 4.07	0.000^#^
FBG (mmol/L)	6.31 ± 2.32	6.06 ± 1.98	6.58 ± 2.64	0.505
Uric acid (µmol/L)	359.47 ± 100.60	350.84 ± 102.28	368.97 ± 98.67	0.308
eGFR [mL•min^-1^• (1.73 m^2^)^-1^]	97.68 ± 17.90	99.42 ± 17.65	95.77 ± 18.12	0.248^#^
TC (mmol/L)	4.33 ± 1.24	4.41 ± 1.31	4.23 ± 1.17	0.330
TG (mmol/L)	1.82 ± 1.38	1.85 ± 1.30	1.80 ± 1.48	0.380
LDL-C (mmol/L)	2.81 ± 0.99	2.86 ± 1.05	2.76 ± 0.93	0.352
HDL-C (mmol/L)	1.05 ± 0.27	1.05 ± 0.22	1.05 ± 0.31	0.648
Lipoprotein(a) (mg/L)	266.12 ± 253.44	253.71 ± 235.24	279.74 ± 273.29	0.765
Apolipoprotein A1 (g/L)	1.14 ± 0.23	1.17 ± 0.23	1.11 ± 0.23	0.084
Apolipoprotein B (g/L)	0.81 ± 0.28	0.82 ± 0.30	0.79 ± 0.26	0.593

Values are presented as mean ± standard deviation. WBC, white blood cell; PLR, platelet to lymphocyte ratio; FBG, fasting blood glucose; TC, total cholesterol; eGFR, estimated glomerular filtration rate; TG, triglyceride; LDL-C, low-density lipoprotein cholesterol; HDL-C, high-density lipoprotein cholesterol. For continuous variables, “^#^” denotes comparisons made using the Student’s *t*-test, while other variables were compared using the Mann-Whitney U test.

### 3.2 Comparative Analysis of Albumin and PLR Levels Across Various Subgroups

**Fig. 2.**
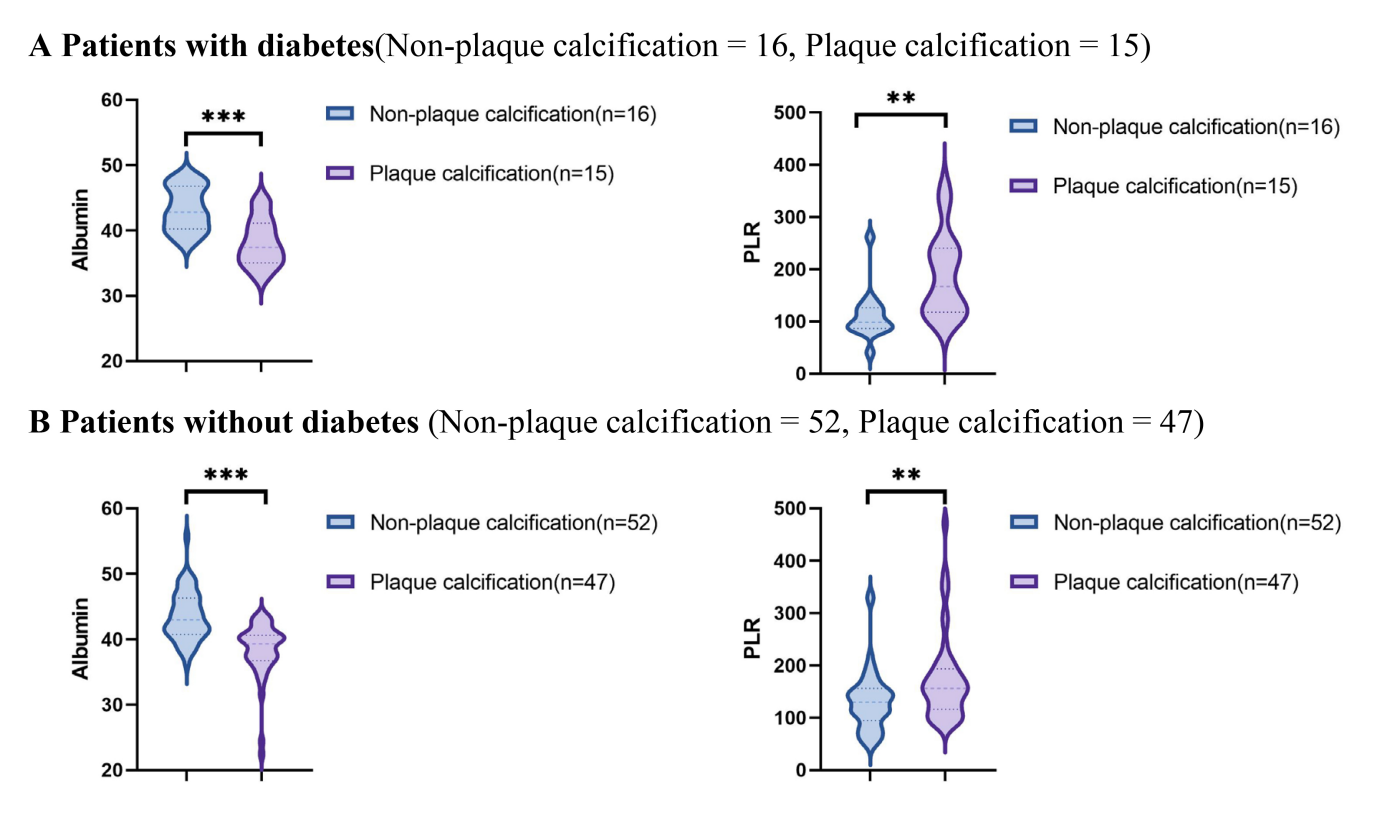
**Relationship between the PLR and albumin and coronary plaque calcification in patients with or without diabetes**. PLR, platelet to lymphocyte ratio; ** *p* < 0.01; *** *p* < 0.001.

**Fig. 3.**
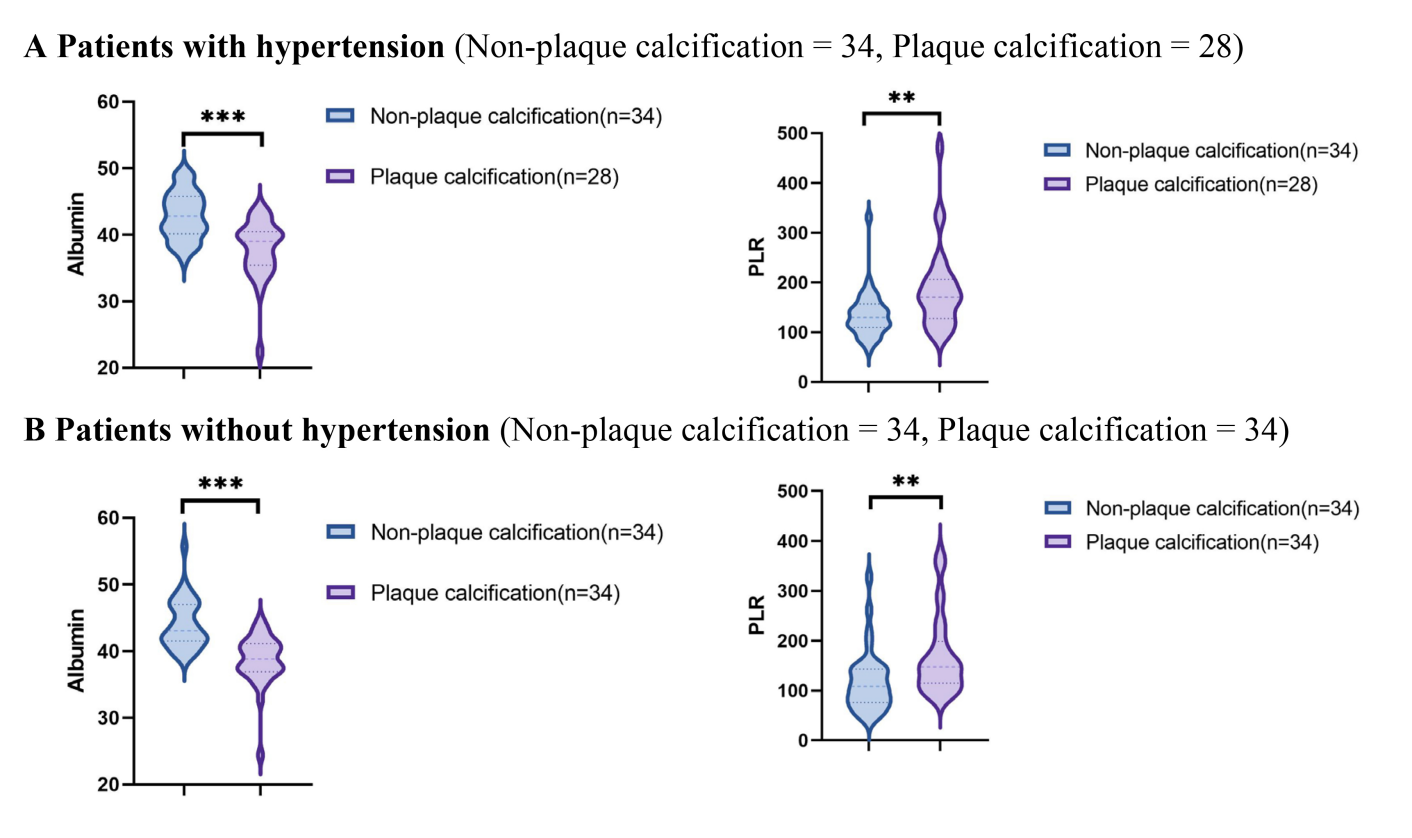
**Relationship between the PLR and albumin and coronary plaque calcification in patients with or without hypertension**. PLR, platelet to lymphocyte ratio; * *p* < 0.05; ** *p* < 0.01; *** *p* < 0.001.

**Fig. 4.**
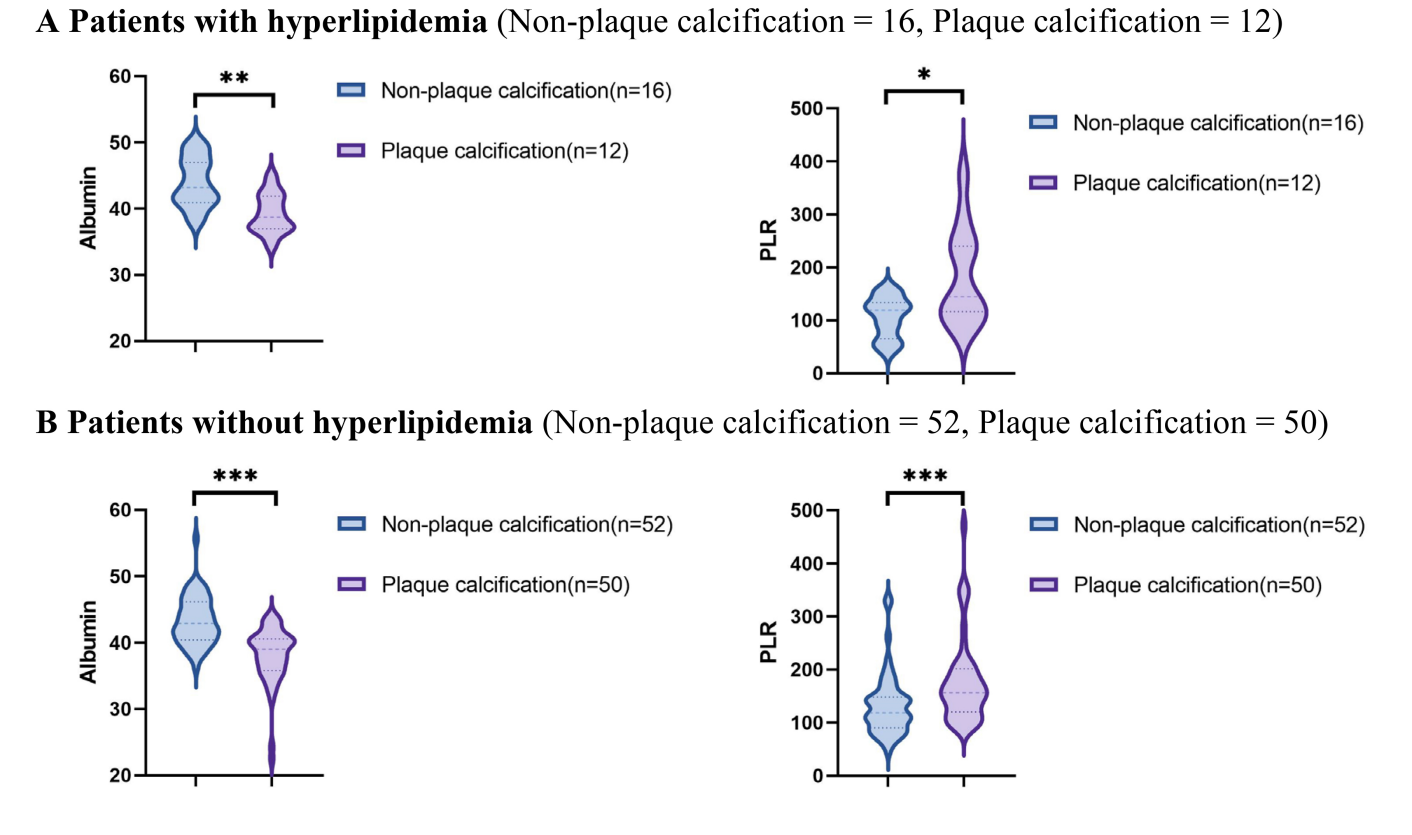
**Relationship between the PLR and albumin and coronary plaque calcification in patients with or without hyperlipidemia**. PLR, platelet to lymphocyte ratio; * *p* < 0.05; ** *p* < 0.01; *** *p* < 0.001.

**Fig. 5.**
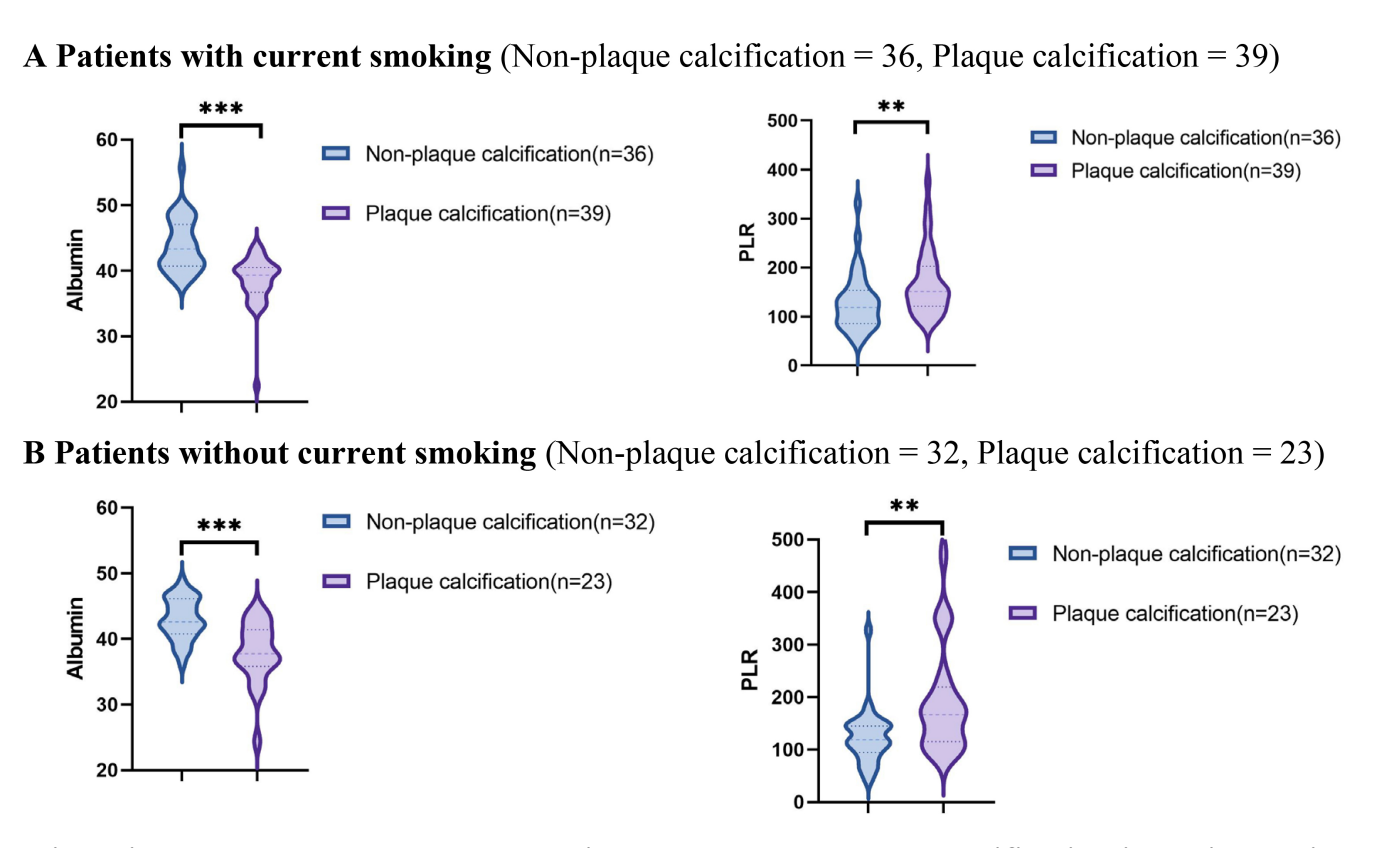
**Relationship between the PLR and albumin and coronary plaque calcification in patients with or without current smoking**. PLR, platelet to lymphocyte ratio; * *p* < 0.05; ** *p* < 0.01; *** *p* < 0.001.

Using data from the presence or absence of plaque calcification along with traditional risk factors such as hypertension, diabetes, hyperlipidemia, and current smoking status, the study population was stratified into distinct subgroups to facilitate a comparison of the PLR and albumin levels between the plaque calcification and non-plaque calcification groups. As illustrated in Figs. 2,3,4,5, the non-plaque calcification group, across various subgroups, exhibited significantly higher levels of albumin in comparison to the plaque calcification group, while the PLR was markedly lower in the non-plaque calcification group than in the plaque calcification group (*p* < 0.05).

### 3.3 Associations of PLR and Albumin with Coronary Artery Plaque Calcification

The results of the Spearman rank correlation analysis examining the relationships between the PLR, albumin, and coronary artery plaque calcification are presented in Table 3. The PLR demonstrated a significant positive correlation with coronary artery plaque calcification (r_s_ = 0.373, *p* < 0.001), whereas albumin exhibited a significant negative correlation (r_s_ = –0.585, *p* < 0.001).

**Table 3.** **Spearman correlation rank test**.

Parameters	r_s_	*p* value
PLR	0.373	*p * < 0.001
Albumin	–0.585	*p * < 0.001

PLR, platelet to lymphocyte ratio.

Employing both univariate and multivariate logistic regression analyses, we investigated the factors predictive of the occurrence of coronary artery plaque calcification in patients with ACS (Table 4). The univariate logistic regression analysis identified several potential predictors of plaque calcification in patients with ACS, including lymphocyte count (odds ratios (OR) 0.468, 95% CI 0.284–0.768, *p* = 0.003), PLR (OR 1.012, 95% CI 1.006–1.019, *p* < 0.001), and albumin levels (OR 0.637, 95% CI 0.539–0.752, *p* < 0.001). Given the strong correlation between lymphocyte count and the PLR (r_s_ = –0.777, *p* < 0.001), lymphocyte count was excluded from the multivariate logistic regression analysis to mitigate collinearity effects. Within the multivariate logistic regression analysis, the PLR (OR 1.011, 95% CI 1.002–1.019, *p* = 0.014) and albumin levels (OR 0.642, 95% CI 0.539–0.764, *p* < 0.001) emerged as independent predictors of coronary artery plaque calcification.

**Table 4.** **Logistic regression analysis of calcification**.

Variables	Univariate	*p* value	Multivariate	*p* value
	OR (95% CI)		OR (95% CI)	
Age	1.000 (0.977, 1.024)	0.994		
Gender	0.804 (0.262, 2.461)	0.702		
BMI	0.950 (0.839, 1.076)	0.423		
Diabetes mellitus	1.037 (0.463, 2.325)	0.929		
Hypertension	0.824 (0.413, 1.642)	0.581		
Hyperlipidemia	0.780 (0.336, 1.812)	0.564		
Family history of CAD	0.627 (0.241, 1.633)	0.339		
Current smoking	1.507 (0.747, 3.040)	0.252		
LDL-C	0.895 (0.630, 1.270)	0.535		
HDL-C	0.992 (0.270, 3.647)	0.990		
Lipoprotein(a)	1.000 (0.999, 1.002)	0.558		
Lymphocyte count	0.468 (0.284, 0.768)	0.003		
PLR	1.012 (1.006, 1.019)	0.000	1.011 (1.002, 1.019)	0.014
Albumin	0.637 (0.539, 0.752)	0.000	0.642 (0.539, 0.764)	0.000

OR, odds ratios; CI, confidence interval; BMI, body mass index; CAD, coronary artery disease; LDL-C, low-density lipoprotein cholesterol; HDL-C, high-density lipoprotein cholesterol; PLR, platelet to lymphocyte ratio.

**Fig. 6.**
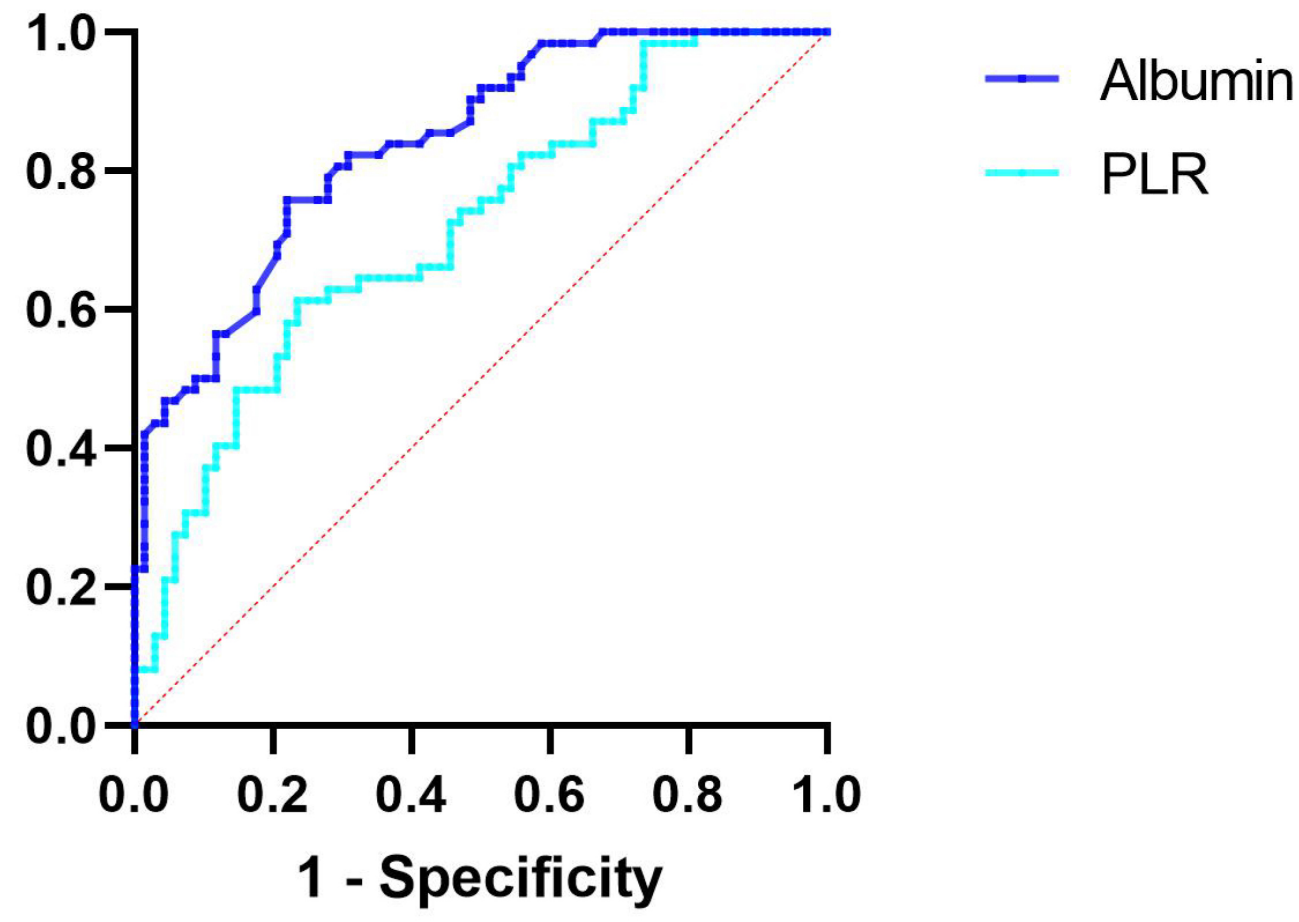
**ROC curve analysis evaluating the predictive ability of albumin and PLR for plaque calcification**. The cutoff value of albumin associated with plaque calcification was albumin <40.65 with 75.8% sensitivity and 77.9% specificity (AUC 0.838, 95% CI 0.773–0.904, *p* < 0.001), while the cutoff value of PLR associated with plaque calcification was PLR >145.04 with 61.3% sensitivity and 76.5% specificity (AUC 0.716, 95% CI 0.628–0.803, *p* < 0.001). ROC, receiver operating characteristic; PLR, platelet to lymphocyte ratio; AUC, area under the ROC curve; CI, confidence interval.

Furthermore, ROC curve analysis revealed that, for patients with ACS, an albumin level of 40.65 g/L serves as the optimal cutoff point associated with the risk of plaque calcification. An albumin level below 40.65 g/L was associated with a sensitivity of 75.8% and a specificity of 77.9% for plaque calcification (AUC = 0.838, 95% CI 0.773–0.904, *p* < 0.001) (Fig. 6).

The optimal threshold associated with the risk of plaque calcification was determined to be a PLR of 145.04. A PLR exceeding 145.04 was associated with a sensitivity of 61.3% and a specificity of 76.5% for plaque calcification (AUC = 0.716, 95% CI 0.628–0.803, *p* < 0.001) (Fig. 6).

## 4. Discussion

Our study aimed to evaluate the potential relationship between prevalent laboratory parameters and coronary artery plaque calcification through the use of OCT. The primary findings of this study are as follows: (1) Irrespective of the presence of traditionally recognized high-risk factors for coronary artery plaque calcification, such as diabetes, hypertension, hyperlipidemia, and smoking, the plaque calcification group exhibited significantly lower levels of albumin compared to the non-plaque calcification group, while the PLR was elevated in the plaque calcification group; (2) Albumin levels exhibited a significant negative correlation with coronary artery plaque calcification in patients with ACS, whereas PLR displayed a positive correlation; (3) In both univariate and multivariate regression analyses, albumin levels and the PLR emerged as independent predictive factors for coronary artery plaque calcification in patients with ACS.

Cardiovascular disease continues to be a predominant cause of global mortality, with nearly half of all fatalities attributed to ischemic heart disease [12]. ACS is recognized as one of the most prevalent cardiovascular conditions encountered in clinical practice and serves as a primary contributor to heart failure (HF). The primary etiology of ACS is attributed to the rupture of atherosclerotic plaques, which subsequently leads to thrombus formation [3]. Identifying vulnerable plaques, which may precipitate coronary artery thrombosis, is essential for the prevention of acute coronary events [11]. Previous studies have demonstrated that CAC functions as an independent predictor of cardiovascular events, correlating significantly with plaque progression and vulnerability [4, 5]. A meta-analysis of 45 studies involving 192,080 asymptomatic and 32,477 symptomatic patients, followed for up to 11 years, demonstrated that CAC is significantly associated with an increased risk of major adverse cardiovascular and cerebrovascular events as well as all-cause mortality [14]. Similarly, in the Mediators of Atherosclerosis in South Asians Living in America (MASALA) study, the 10-year risk of atherosclerotic cardiovascular disease in South Asians was strongly correlated with the CAC burden [15]. CAC also demonstrates a significant association with both stent thrombosis and in-stent restenosis [16, 17, 18]. Additionally, calcium ions have the potential to disrupt the polymer coating of drug-eluting stents, thereby impairing the effective delivery of therapeutic agents to the vessel wall [19, 20]. The Multi-center Prospective Study to Evaluate Outcomes of Moderate to Severely Calcified Coronary Lesions (MACE Trial) [21] investigated the influence of lesion plaque calcification on PCI outcomes. The study found that severe plaque calcification significantly affected lesion success rates (83.3% vs. 94.7% for none/mild plaque calcification) and was associated with elevated 1-year major adverse cardiac events (MACE) rates (24.4% vs. 4.7% for none/mild plaque calcification). Therefore, investigating factors associated with the development of CAC and implementing targeted preventive measures to mitigate the progression of atherosclerosis is of paramount clinical significance in reducing readmission and mortality rates among patients with ACS. Although coronary angiography is extensively employed to evaluate the extent and severity of arterial narrowing, its sensitivity in detecting calcified plaques is limited, particularly for smaller lesions. OCT, in contrast, functions as a high-resolution cross-sectional intracoronary imaging technique, facilitating the clear identification of internal coronary artery structures and plaque characteristics. OCT has the potential to delineate the intricate details of CAC [10, 11]. This study investigated the potential relationship between common laboratory parameters and coronary artery plaque calcification utilizing OCT techniques.

This study found that, independent of traditional risk factors for plaque calcification—such as hypertension, diabetes, hyperlipidemia, and current smoking status—albumin levels in the non-plaque calcification group were significantly higher than those in the plaque calcification group, whereas the PLR was significantly lower in the latter group. Furthermore, albumin levels demonstrated a significant negative correlation with coronary artery plaque calcification in ACS patients, whereas PLR exhibited a positive correlation. Notably, both factors emerged as independent predictors of coronary artery plaque calcification in this patient population.

During the processes of inflammation and atherosclerosis, lymphocytes are recognized for their protective role [22]. Lymphopenia represents a prevalent hematological manifestation in ACS patients and is associated with an elevated risk of adverse outcomes in individuals undergoing coronary angiography [23, 24]. PLR, calculated by dividing the platelet count by the lymphocyte count, is regarded as a predictive biomarker of inflammation and adverse outcomes in various cardiovascular diseases. This measurement encompasses both inflammatory and thrombotic pathways, potentially providing superior prognostic value compared to platelet or lymphocyte counts alone [22]. In a prospective longitudinal study involving 799 patients with acute myocardial infarction (AMI) who underwent successful primary PCI within 12 hours of the onset of chest pain [25], the incidence of MACE in the high PLR group was approximately 2.8 times greater than that observed in the low PLR group. Furthermore, a study utilizing data from the Intensive Care Medical Information Market III database [26] indicated that a high PLR was associated with an elevated short-term mortality risk in critically ill patients with non-ST-segment elevation ACS. Additionally, a high PLR was strongly associated with the no-reflow phenomenon following vascular reconstruction, serving as an independent risk factor for inadequate cardiac reperfusion and ineffective saphenous vein grafting [27, 28, 29]. As a marker of inflammation, the PLR has demonstrated significant predictive value in the fields of oncology, hematology, immunology, and cardiovascular diseases [30].

The prevalence of hypoalbuminemia varies from 13% in patients with stable coronary artery disease to between 20% and 30% in those with ACS and myocardial infarction [31]. The occurrence of ischemic heart disease, HF, AF, stroke, and venous thromboembolism is inversely correlated with serum albumin levels, indicating that lower serum albumin levels are associated with a higher incidence of these conditions [31]. Hypoalbuminemia serves as a strong prognostic indicator in patients with CAD [32]. After adjusting for the severity of CAD, ejection fraction, BMI, and levels of inflammation, low serum albumin concentration significantly correlates with all-cause mortality and the incidence of adverse cardiovascular events [33, 34]. The Framingham Offspring Study, a 22-year follow-up involving 4506 participants, identified serum albumin as an independent predictor of an initial MI [35]. In a study involving 341 patients undergoing PCI [36], hypoalbuminemia was found to be associated with in-stent restenosis. Hypoalbuminemia typically results from reduced hepatic synthesis, increased catabolic metabolism, enhanced vascular permeability, and losses occurring in the kidneys and intestines [37]. Malnutrition and inflammation are widely recognized as primary contributors to the development of hypoalbuminemia [31]. Hypoalbuminemia typically arises from diminished hepatic synthesis, elevated catabolic metabolism, increased vascular permeability, and losses occurring in the kidneys and intestines [37]. Malnutrition and inflammation are widely considered to be primary contributors to the occurrence of hypoalbuminemia [31]. An OCT study investigating the relationship between malnutrition and vulnerable plaques revealed that malnutrition is a predictor of vulnerable plaques and is closely associated with inflammatory progression [38]. The close association with plaque calcification may be attributed to the body’s inflammatory levels. The causal link between inflammation and plaque calcification has been clinically established by positron emission tomography scans [39]. The PLR serves as a derivative indicator associated with inflammation, whereas albumin exhibits anti-inflammatory, antioxidant, anticoagulant, and antiplatelet aggregation properties. Inflammation is widely regarded as a primary contributor to the occurrence of hypoalbuminemia [31]. Inflammation can stimulate the expression of Runt-related transcription factor 2 (Runx2), which plays a pivotal role in the process of plaque calcification. Runx2 and BMP (bone morphogenetic protein) collaboratively facilitate the differentiation of vascular smooth muscle cells (VSMCs) into osteoblast-like cells [40]. The latter promotes arterial plaque calcification through several pathways, including hydroxyapatite production, the release of cell membrane microvesicles and extracellular vesicles that downregulate mineralization inhibitors, and the enhancement of extracellular matrix deposition in the vascular wall, along with the activation and generation of matrix metalloproteinases [41]. Furthermore, Runx2 can upregulate the expression of receptor activator of nuclear factor-κB ligand (RANKL) and bind to its corresponding receptor, RANK. This interaction creates a microenvironment that is conducive to the expression of tissue-nonspecific alkaline phosphatase (TNAP), thereby facilitating phosphate mineralization [40]. In the context of widespread inflammation, macrophage-derived vesicles are released within the necrotic core of atherosclerotic plaques, serving as nucleation sites for plaque calcification. In the presence of persistent inflammation, a cyclical process of macrophage infiltration and repair occurs through plaque calcification. This finding is consistent with the results of our study, demonstrating a positive correlation between the PLR and macrophage infiltration in plaques (r_s_ = 0.254, *p* = 0.004). We hypothesize that albumin and PLR, readily accessible parameters, possess predictive value for coronary artery plaque calcification, which is essential for guiding the prevention of adverse cardiovascular events. Nevertheless, further research is required to validate our hypothesis.

## 5. Limitations

This study has several limitations. First, this investigation is a single-center retrospective cross-sectional study with a limited sample size; therefore, caution should be exercised when generalizing the findings to all patients. Second, the majority of the study population in this research consisted of males (89.2%); thus, the findings may not fully reflect changes in female patients. Third, we did not evaluate the dynamic changes in the PLR and albumin; rather, we assessed only the spot values. Consequently, we cannot ascertain whether PLR and albumin continue to serve as predictive indicators of coronary artery plaque calcification in ACS patients. Lastly, the impact of plaque calcification may exhibit a biphasic nature; plaque rupture has been demonstrated to be positively correlated with the quantity of punctate plaque calcifications while negatively correlated with the amount of larger plaque calcifications. Punctate plaque calcification represents the early active stage of vascular plaque calcification associated with inflammation, whereas large plaque calcifications indicate a relatively inactive late stage. This study did not distinguish between punctate and large plaque calcifications; rather, it provided only a qualitative assessment of the presence or absence of plaque calcification. Therefore, to gain a deeper understanding of the relationship between the PLR, albumin, and plaque calcification in coronary artery plaques of ACS patients, large-scale prospective cohort studies and more comprehensive subgroup analyses are required.

## 6. Conclusions

Albumin and the PLR are cost-effective and readily accessible laboratory parameters, which exhibit significant associations with coronary artery plaque calcification in patients with ACS. Both markers have been identified as independent predictors of coronary artery plaque calcification, underscoring their potential utility in routine clinical practice for cardiovascular risk assessment. Future prospective studies involving larger, multicenter cohorts are crucial to validate these findings and determine their applicability across diverse patient populations. Investigations into the mechanisms linking these biomarkers to plaque calcification could potentially unveil novel therapeutic targets.

## Availability of Data and Materials

The datasets utilized or analyzed during the current study are available from the corresponding author upon reasonable request.
